# HB-EGF Plasmatic Level Contributes to the Development of Early Risk Prediction Nomogram for Severe COVID-19 Cases

**DOI:** 10.3390/biomedicines12020373

**Published:** 2024-02-05

**Authors:** Alexandra Ioana Moatar, Aimee Rodica Chis, Diana Nitusca, Cristian Oancea, Catalin Marian, Ioan-Ovidiu Sirbu

**Affiliations:** 1 Doctoral School, University of Medicine and Pharmacy “Victor Babes”, 300041 Timisoara, Romania; moatar.alexandra@umft.ro (A.I.M.); nitusca.diana@umft.ro (D.N.); 2 Department of Biochemistry, University of Medicine and Pharmacy “Victor Babes”, 300041 Timisoara, Romaniaovidiu.sirbu@umft.ro (I.-O.S.); 3 Center for Complex Network Science, University of Medicine and Pharmacy “Victor Babes”, 300041 Timisoara, Romania; 4 Department of Pneumology, University of Medicine and Pharmacy “Victor Babes”, 300041 Timisoara, Romania

**Keywords:** HB-EGF, prediction marker, COVID-19 severe, clinical correlations

## Abstract

(1) Background: Heparin-Binding Epidermal Growth Factor-like Growth Factor (HB-EGF) is involved in wound healing, cardiac hypertrophy, and heart development processes. Recently, circulant HB-EGF was reported upregulated in severely hospitalized COVID-19 patients. However, the clinical correlations of HB-EGF plasma levels with COVID-19 patients’ characteristics have not been defined yet. In this study, we assessed the plasma HB-EGF correlations with the clinical and paraclinical patients’ data, evaluated its predictive clinical value, and built a risk prediction model for severe COVID-19 cases based on the resulting significant prognostic markers. (2) Methods: Our retrospective study enrolled 75 COVID-19 patients and 17 control cases from May 2020 to September 2020. We quantified plasma HB-EGF levels using the sandwich ELISA technique. Correlations between HB-EGF plasma levels with clinical and paraclinical patients’ data were calculated using two-tailed Spearman and Point-Biserial tests. Significantly upregulated parameters for severe COVID-19 cases were identified and selected to build a multivariate logistic regression prediction model. The clinical significance of the prediction model was assessed by risk prediction nomogram and decision curve analyses. (3) Results: HB-EGF plasma levels were significantly higher in the severe COVID-19 subgroup compared to the controls (*p* = 0.004) and moderate cases (*p* = 0.037). In the severe COVID-19 group, HB-EGF correlated with age (*p* = 0.028), pulse (*p* = 0.016), dyspnea (*p* = 0.014) and prothrombin time (PT) (*p* = 0.04). The multivariate risk prediction model built on seven identified risk parameters (age *p* = 0.043, HB-EGF *p* = 0.0374, Fibrinogen *p* = 0.009, PT *p* = 0.008, Creatinine *p* = 0.026, D-Dimers *p* = 0.024 and delta miR-195 *p* < 0.0001) identifies severe COVID-19 with AUC = 0.9556 (*p* < 0.0001). The decision curve analysis revealed that the nomogram model is clinically relevant throughout a wide threshold probability range. (4) Conclusions: Upregulated HB-EGF plasma levels might serve as a prognostic factor for severe COVID-19 and help build a reliable risk prediction nomogram that improves the identification of high-risk patients at an early stage of COVID-19.

## 1. Introduction

COVID-19 manifests through various clinical forms, ranging from paucisymptomatic to severe respiratory distress syndrome [1]. This disease shows a high recovery rate, but its long-term health effects develop frequently in survivors diagnosed with moderate and severe forms of the disease [2]. Multiple risk factors such as age, sex, associated comorbidities, and body weight were linked to the severity of the disease. However, little is known about the biological mechanisms involved in the aggravation of the disease and its prognostic biomarkers.

Growth factors have been considered essential players in the pathogenesis of COVID-19 [3]. SARS-CoV-2 infection is associated with plasma release of cytokines and growth factors, mediating coagulation dysfunctions and tissue injury that contribute to a severe disease outcome [4]. Among these growth factors, Heparin-binding EGF-like growth factor (HB-EGF) is expressed in many organs, playing an essential role in tissue regeneration and repair throughout the body [5]. HB-EGF is synthesized as a type I membrane-anchored protein (pro-HB-EGF), which, after extensive proteolytic processing by matrix metalloproteinases, is converted to the soluble, mature form (sHB-EGF). The active protein binds to EGFR (epidermal growth factor receptors) and modulates cellular processes such as cell adhesion, motility, and angiogenesis [6]. HB-EGF is involved in a wide variety of pathological processes, such as wound healing [7], cardiac hypertrophy [8], pulmonary fibrosis, oncogenic transformation, and hypertension [9]. Infections with respiratory viruses contribute to a wide range of lung diseases and complications ranging from asthma to pulmonary fibrosis [10]. These mechanisms depend on epidermal growth factor receptor ligands such as amphiregulin (AREG) and HB-EGF released from virus-infected epithelium cells and inflammatory cells [11]. Consequently, it was suggested that respiratory viruses might co-opt epidermal growth factor receptors and their activating ligands to promote such pathogenetic mechanisms. On the other hand, in influenza viral infections, it was reported that HB-EGF mediated the internalization of epidermal growth factor receptors, thus pointing to a novel anti-viral strategy [12]. In mice, the SARS-CoV virus triggers upregulation of HB-EGF levels and is associated with lung damage [13]. In humans, SARS-CoV-2 infection is associated with a significant increase in serum HB-EGF levels in patients with the severe form of the disease [14]. However, it is unclear whether the HB-EGF upregulation in severe COVID-19 is a molecular trigger or a simple bystander of the disease severity. 

Here, we quantified the expression of early (first two days upon hospital admission) plasma HB-EGF in COVID-19 patients, evaluated its correlations with known risk factors, and developed a predictive model for disease severity.

## 2. Materials and Methods

### 2.1. Patients

Our retrospective study enrolled 75 COVID-19 patients (diagnosed based on a positive quantitative real-time polymerase chain reaction (qRT-PCR) test for SARS-CoV-2 infection) and 17 control cases without SARS-CoV-2 infection, admitted to the Clinical Hospital of Infectious Diseases and Pneumophysiology (CHIDP), Timisoara, Romania, between May 2020 and September 2020. All cases were managed according to the clinical guidelines in use at the time [15]. The inclusion criteria for the respondents in the COVID-19 group were a laboratory-confirmed SARS-CoV-2 infection as determined by qRT-PCR, admission to the hospital with suggestive symptoms of COVID-19 disease, and the willingness of the subjects to provide written informed consent. The exclusion criteria for the respondents were a negative qRT-PCR test for SARS-CoV-2 infection, refusal to participate in the current study, and incomplete clinical and paraclinical data. Patients were assigned to the severe COVID-19 group if they required oxygen supplementation with or without non-invasive ventilation or mechanical ventilation and if they died. The moderate group included all patients without the severe disease criteria. The current study was approved by the Ethics Committee of Victor Babes University of Medicine and Pharmacy no. 34/28.07.2020, Timisoara, Romania, and was carried out in accordance with the principles of the Declaration of Helsinki. All patients were informed and provided written consent, recorded as such in their medical records. 

### 2.2. Plasma Collection

The blood samples were collected in EDTA (ethylenediaminotetraacetic acid)-coated vacutainers within the first two days upon hospital admission, before the onset of COVID-19 specific therapy (interferon-beta, Kaletra, Tocilizumab, Corticosteroids, Antibiotics). All blood samples were processed (centrifugation at 1500× *g* for 10 min) within three hours after collection, and plasma was stored and aliquoted at −80 °C until further use.

### 2.3. Clinical Data Collection

The present study expands on a previous work in our lab, which investigated plasma hsa-miR-195 in COVID-19 patients [16]. The patients’ demographic, clinical, and paraclinical characteristics from the day of admission to the hospital were collected from CHIDP electronic medical records. All data were stored anonymized in the Biochemistry Department server (Supplementary File S1).

### 2.4. HB-EGF Quantification

Human HB-EGF was measured in 100µL plasma samples using the HB-EGF Human ELISA kit (INVITROGEN, catalog number EHHBEGF, 96 tests, Waltham, MA, USA), according to the manufacturer’s protocol. The optical densities were read in duplicate at 450 nm and recorded on a GloMax^®^ Discover Microplate Reader device (Promega, Madison, WI, USA). Standards were serially diluted between 16.38 pg/mL and 4000 pg/mL range with Reagent Diluent and assayed in duplicate according to the manufacturer’s instructions. The specificity of the ELISA kit was proved through the non-cross-reactivity with a wide range of cytokines tested: human Angiogenin, BDNF, BLC, ENA-78, FGF-4, IL-1 alpha, IL-1 beta, IL-2, IL-3, IL-4, IL-5, IL6, IL-7, IL-8, IL-9, IL-11, IL-12 p70, IL-12 p40, IL-13, IL-15, I309, IP-10, G-CSF, GM-CSF, IFN-gamma, Leptin (OB), MCP-1, MCP-2, MCP-3, MDC, MIP-1 alpha, MIP-1 beta, MIP-1 delta, PARC, PDGF, RANTES, SCF, TARC, TGF-beta, TIMP-1, TIMP2, TNF-alpha, TNF-beta, TPO, VEGF. The sensitivity of detecting human HB-EGF was estimated to be around 20 pg/mL. HB-EGF concentrations in the patient’s samples were interpolated based on the standard curve constructed by plotting the mean absorbance against the known concentrations of standards for each assay.

### 2.5. Statistics

The statistical analyses were conducted using GraphPad Prism version 9.5.0 software for Windows (GraphPad Software, Inc., San Diego, CA, USA) and R4.3.1 software [17]. Shapiro–Wilk test was used to verify the data distribution normality. Descriptive statistics (mean, standard deviations, median, interquartile range, and percentage) were calculated for the patients’ demographic, clinical, and paraclinical characteristics. Inter-group clinical data comparisons were performed using Fisher’s exact test for categorical variables and the two-tailed Mann–Whitney U test for continuous ones. Plasma biomarkers differentially expressed (*p* < 0.05) in severe COVID-19 were further considered as predictive candidates for severity, and correlation analyses were conducted using the two-tailed Spearman test for continuous variables and the Point-Biserial test for continuous versus binary variables. All severe COVID-19 predictive candidates were subjected to multiple logistic regression analyses leading to a prediction model for which the Receiver Operating Characteristics (ROC) curve (using the Wilson/Brown method) and the Hosmer–Lemeshow test (to find the goodness fit of the model) were calculated. To assess the accuracy and performance of the predictive risk model, we calculated the Youden Index with the formula Sensitivity + Specificity-1 and the optimal threshold on the ROC curve. The prediction risk nomogram was designed according to the final logistic regression model. To evaluate the nomogram’s predictive reliability, we calculated Harrell’s C-index and performed a calibration curve. The nomogram was internally validated by using the bootstrap method with 1000 resamples. Finally, a decision curve analysis was performed to assess the clinical net benefit of the risk prediction nomogram. The risk prediction nomogram and the decision curve analysis were designed using the “rms”, “tidyverse”, and “survival” R packages.

## 3. Results

### 3.1. Patients’ Characteristics

The demographic, clinical, and paraclinical data of COVID-19 patients enrolled in this study were summarized in Table 1. Compared to moderate cases, the severe COVID-19 patients were older (*p* = 0.0043) and showed more frequently associated pathologies like hypertension (*p* = 0.046), cardiovascular (*p* = 0.005) and oncologic (*p* = 0.015) pathologies. The molecular investigations revealed significantly upregulated levels of, fibrinogen (*p* = 0.009), PT (*p* = 0.008), creatinine (*p* = 0.026), D-Dimers (*p* = 0.024) and HB-EGF (*p* = 0.037) for the severe cases. As mentioned, these patients have been previously investigated for hsa-miR-195 expression in plasma and the delta miR-195 (microRNA-195 normalized to cel-miR-39) was significantly increased (*p* < 0.0001). 

Clinically, severe COVID-19 patients resulted to be more often dyspneic (*p* = 0.0006), complain of anosmia (*p* = 0.0251), and thoracic pain (*p* = 0.0005) (Table 2). As expected, severe COVID-19 patients showed more frequent thoracic CT scan anomalies like large glass opacities (*p* = 0.0422), large consolidate opacities (*p* = 0.0079), and diffuse infiltrate (*p* = 0.0141) (Table 2).

### 3.2. HB-EGF Quantification Results

**Figure 1:**
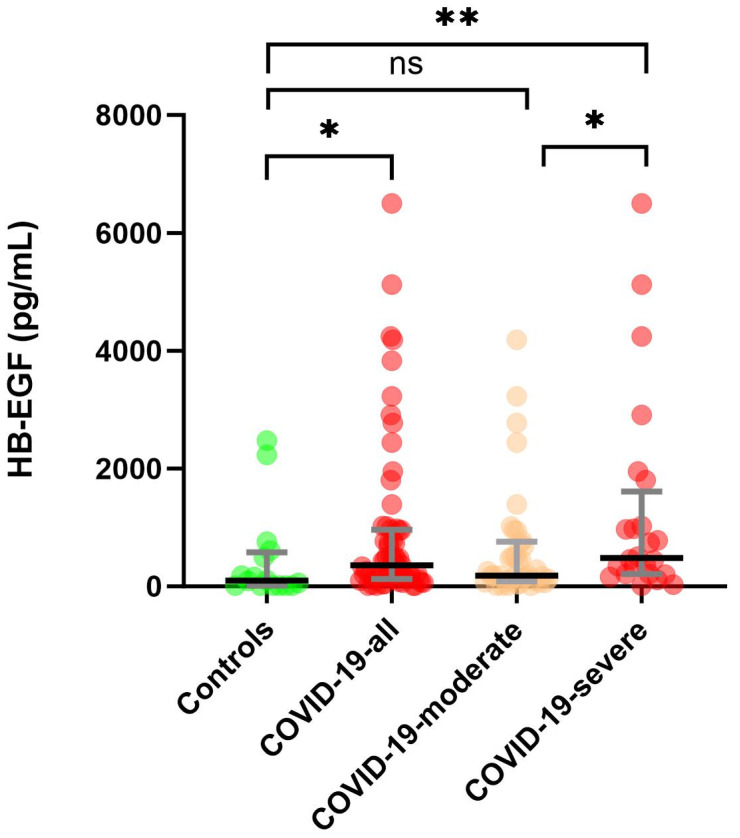
Scatter dot plot with median (IQR) representation of HB-EGF plasma (pg/mL) concentrations in the four cohorts (Controls, COVID-19-all, COVID-19-moderate, and COVID-19-severe). Stars represent the level of significance between the groups: * < 0.05, ** < 0.01; ns—not significant.

Overall, HB-EGF levels in plasma were significantly higher in COVID-19 total patients and COVID-19 severe group compared to the control group (*p* = 0.0306 and *p* = 0.0049, respectively) (Figure 1). COVID-19 severe plasma HB-EGF levels were significantly higher (*p* = 0.0371) compared to moderate cases. However, there was no significant difference between the moderate COVID-19 cases and the controls.

### 3.3. HB-EGF Correlations

**Figure 2:**
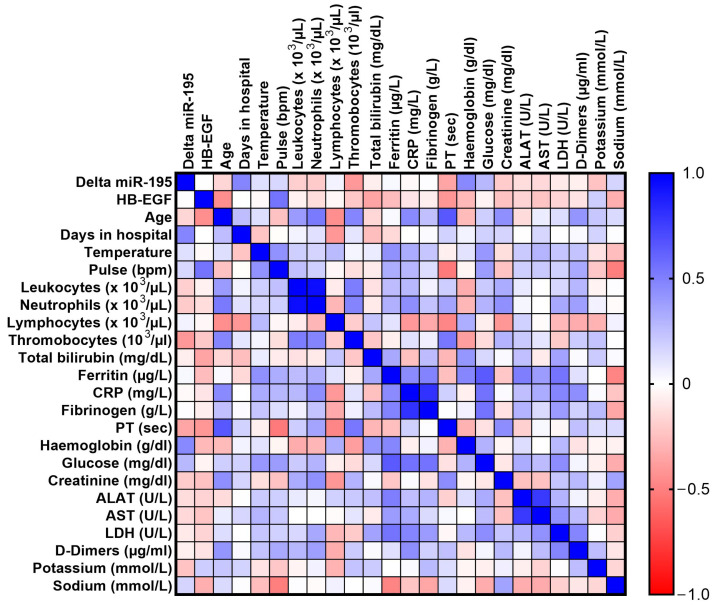
Spearman correlation matrix of HB-EGF plasma levels with the paraclinical parameters of COVID-19 severe cases. The colors represent the strength of correlation, dark blue signifying a strong positive correlation, while dark red a strong negative correlation.

The results of the Spearman correlation analysis for severe COVID-19 patients were plotted as a heatmap in Figure 2. HB-EGF plasma levels positively correlated with pulse (*p* = 0.016, *r* = 0.542) and negatively correlated with age (*p* = 0.028, *r* = −0.44) and PT (*p* = 0.041, *r* = −0.410). Point-Biserial test of dichotomous variables showed that HB-EGF negatively correlates with dyspnea (*p* = 0.014, *r* = −0.481) and sore throat (*p* = 0.0276, *r* = −0.480). In moderate COVID-19 and all COVID-19 cohorts, HB-EGF did not significantly correlated with any of these parameters. The entire correlation analysis is presented in Supplementary File S2.

### 3.4. Selection of the Risk Prediction Biomarkers

We used univariate descriptive analysis to select (*p* < 0.05) the potential risk biomarkers suitable for inclusion in the multivariate logistic model for prediction of COVID-19 severity. HB-EGF (*p* = 0.0371), delta miR-195 (*p* < 0.0001), PT (*p* = 0.0088), fibrinogen (*p* = 0.0094), D-Dimers (*p* = 0.0242), creatinine (*p* = 0.0269) were all significantly increased in the COVID-19 severe group. Since the risk of COVID-19 severity increases due to the isolated effect of increasing age by year [18], we also included age (*p* = 0.0043) in our risk prediction model.

### 3.5. COVID-19 Severity Prediction Risk Model, Nomogram Validation, and Decision Curve Analysis

**Figure 3:**
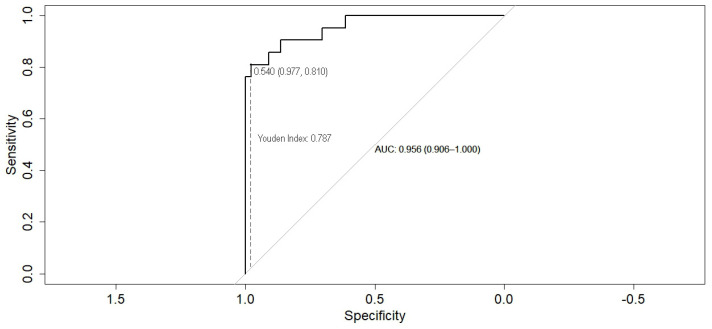
The receiver operating characteristic (ROC) curve (black line) of the risk prediction model plotted with the optimal threshold of 0.540 (representing the value closest to the upper left corner of the ROC curve–grey line) and calculated Youden Index of 0.787 (describing the longest dash vertical line between the ROC curve and the diagonal line). Both indexes are characterized by the maximum values of sensitivity 0.810 and specificity 0.977.

The prediction model was developed through multivariate logistic regression analysis; the results were summarized in Table 3. In the prediction model, delta miR-195 (*p* = 0.0005) and HB-EGF (*p* = 0.0374) remained the only significant independent parameters. The Hosmer–Lemeshow goodness-of-fit test has a high *p*-value (0.9697), indicating a very good logistic regression model fit. As shown in Figure 3, the receiver operating characteristic (ROC) curve of the model had an excellent area under curve (AUC) of 0.9556, with *p*-value less than 0.0001, a negative predictive power of 89.47%, and a positive predictive power of 91.30%. The calculated Youden Index for the ROC curve was 0.787 (at a sensitivity of 0.810 and a specificity of 0.977).

**Figure 4:**
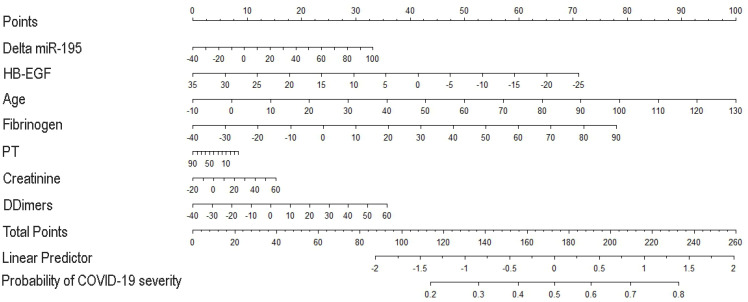
Predictive risk nomogram derived from the final multivariable prognostic model suitable for early identification of COVID-19 severe cases. The predicted probability of COVID-19 severity can be read in two steps: (1) a vertical line from each variable axis can be drawn to the Points axis in order to get the number of each variable; (2) adding the points of all variables, the total point number is obtained which can be mapped into the predicted probability of COVID-19 severity.

Based on the abovementioned variables of the prediction model, we constructed a nomogram for assessing the clinical outcome of the disease (Figure 4), in which each parameter was assigned a point according to each patient’s molecular signature, and the total points corresponded to the patient severity probability. Age resulted in having the most significant regression coefficient, and its range corresponded vertically to the point range 0–100 of the point scale. The proposed nomogram presented a Harell’s C-index of 0.8137 with a 95% CI of 0.7211 to 0.9063, indicating the model had a good predictive ability.

**Figure 5:**
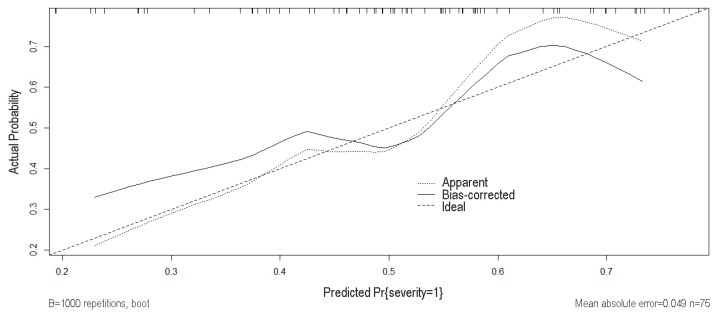
The calibration plot for the prediction risk nomogram. The 45-degree dotted line represents the ideal prediction, the small, dotted line represents the nomogram we constructed, and the dark solid line is the bias-corrected fitted line of the nomogram using the bootstrap internal validation method. The closer the dark solid line is to the ideal line, the better the calibration of the nomogram.

With a mean squared error of 0.0032 and a mean absolute error of 0.049, the calibration plot revealed that the predictions of COVID-19 severity were in good agreement with the observed predictions of this disease severity (Figure 5).

**Figure 6:**
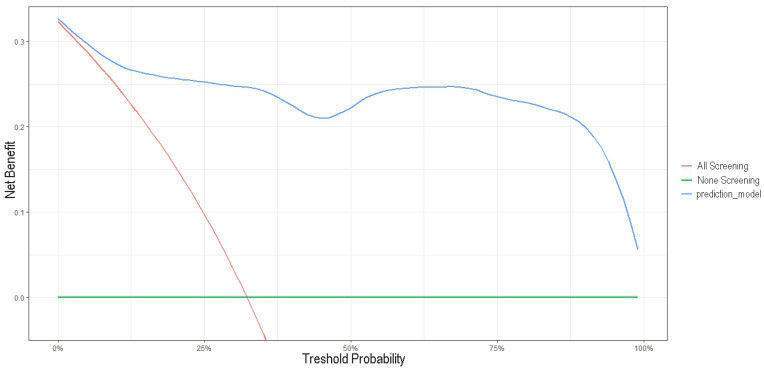
Decision curve analysis of the predicted nomogram. The *x*-axis indicates the threshold probability of the COVID-19 severe outcome, and the *y*-axis measures the net benefit. The red line represents the assumption that all patients are predicted to have a severe outcome of the disease. The green line represents the assumption that none of the patients were predicted to have a severe outcome. The blue line indicates the net benefit of the risk prediction model at different threshold probabilities.

We performed a decision curve analysis to determine whether the benefits of a clinical decision based on the risk prediction model outweigh the disadvantages. The clinical utility of the prediction model is represented by the net benefit illustrated in the graph through the blue line. In our risk prediction model, the net clinical benefit resulted in values over 0.05 and the highest AUC across a wide range of threshold probabilities, from 0.01% to approximately 100%. Therefore, screening COVID-19 patients based on our proposed prediction model would lead to a higher clinical net benefit than all-screening and non-screening strategies. Using this prediction model to determine whether the patients are at high risk of developing a severe form of COVID-19 disease would improve clinical management and might prevent a severe outcome for these patients (Figure 6).

## 4. Discussion

Our data showed a significant early (first two days post-admission) upregulation of plasma HB-EGF in severe COVID-19 patients, similar to serum data reported by de Morais Batista et al. [14]. This suggests that the early physio-pathological mechanisms underlying SARS-CoV-2 infection of human cells might determine HB-EGF upregulation through mechanisms that remain to be determined. Of note, a transient increase in HB-EGF was described in human primary lung microvascular endothelial cells on day 2 post-infection with SARS-CoV-2 [19]. HB-EGF binds to and activates epidermal growth factor receptor (EGFR), a key regulator of lung fibrosis after SARS-CoV-2 infection [20]. Activation of EGFR triggers the EGFR-MAPK signaling axis through a mechanism that also involves the ACE2 receptor [21]. Equally important, Raf/MAPK/Erk signaling pathway was reported to be transiently activated in the very early phase of SARS-CoV-2 infection [22]. Activation of Raf/MAPK/Erk pathway through the EGF receptors is a common cellular response mechanism to various stress stimuli, including viral infections [23,24]. It is worth mentioning that in patients with idiopathic pulmonary fibrosis and mice with induced pulmonary fibrosis, monocytes-derived alveolar macrophages and alveolar epithelial cells are characterized by augmented HB-EGF expressions [25]. Specifically, in vitro results on alveolar epithelial cells revealed that HB-EGF promotes pulmonary fibrosis by activating the fibroblasts migration without altering the gene expression matrix of either the pro-fibrotic genes or the anti-fibrotic genes [25].

Accumulating evidence identified the heart as a direct target of SARS-CoV-2 infection [26,27,28]. SARS-CoV-2 viral proteins have been identified in the cardiomyocytes of COVID-19 without any clinical signs of cardiac involvement [29]. Moreover, upon infection with the SARS-CoV-2 virus, human pluripotent cell-derived cardiomyocytes (hPSC-CMs) showed significant transcriptome changes, with HB-EGF gene expression upregulation when exposed to even middle (0.01) multiplicity of infection rate [30]. In injured cardiac tissue, HB-EGF induced the accumulation of myofibroblasts and macrophages, promoting cardiac hypertrophy [31]. HB-EGF promotes heart fibrosis, and the proliferation of fibroblasts from cardiac tissue occurs via activation of the Akt/mTOR/p10s6k pathway [32]. In turn, molecules such as mTOR (mammalian target of rapamycin) and protein kinase B (PKB, also known as Akt) were used by the SARS-CoV-2 virus for the synthesis of its own viral particles in infected cells [33].

In severe COVID-19 patients, plasma HB-EGF levels correlated with the paraclinical and clinical variables. HB-EGF levels positively correlated (*r* = 0.542, *p* = 0.016) with increased pulse rate and negatively correlated with symptoms such as dyspnea (r = −0.481, *p* = 0.014) and sore throat (*r* = −0.480, *p* = 0.0276) at COVID-19 severe patients. Interestingly, upregulated HB-EGF levels negatively correlated with the age of patients (*r* = −0.44, *p* = 0.028) and PT (*r* = −0.410, *p* = 0.041), both well-known severity predictors in COVID-19. PT, also known as the time of prothrombin, is a coagulation marker that refers to the blood clotting time [34]. Several reports highlighted the clinical relevance of PT in COVID-19 infection, along with other clotting-related parameters such as platelet count, D-Dimers, and fibrinogen [35,36,37,38]. In glioblastoma, HB-EGF acts as a mediator of coagulation activation, a process triggered by hypoxia exposure of these cells [39].

Finally, we built a risk prediction model for COVID-19 severity based on age and the significantly elevated early biomarkers: delta miR-195, HB-EGF, fibrinogen, PT, creatinine, and D-Dimers. This prognostic model had a very good prediction accuracy of the fitted model with a *p*-value of 0.9697 in the Hosmer–Lemeshow test. To further assess the clinical significance of the risk prediction model, we constructed a nomogram and a decision curve. Our nomogram was internally validated through the bootstrap resampling method, C-index calculations, and calibration plot. All these validation methods indicate a good performance of the risk prediction nomogram, characterized by a C-index of 0.8137 (95% CI, 0.7211–0.9063) and an outstanding AUC of 0.9556 (95% CI, 0.9063–1.000). Since the beginning of the pandemic, numerous prognostic nomograms generally based on clinical characteristics, CT scans, and routine laboratory tests were built in an attempt to predict COVID-19 severity, mortality, and survival [40,41,42,43,44,45,46]. Yao et al. developed a risk predictive nomogram for COVID-19 severity based on routine laboratory parameters as CRP (C-reactive protein), ESR (erythrocyte sedimentation rate), LDH (lactate dehydrogenase), age and the Charlson comorbidity score [47]. The predictive nomogram was validated in three cohorts. With AUC values in all three datasets below 0.9. Shi et al. (2023) constructed a predictive nomogram for COVID-19 mortality on multiethnic patients by using routine laboratory parameters and the Asian race criterion, which proved to be an independent risk factor for the outcome [42]. The nomogram was externally validated, with AUC values of 0.860 and 0.847 for the training and validation datasets, respectively. Chen et al. (2020) used a nationwide cohort to build an overall survival prognostic model that included age, comorbidities, dyspnea, and oxygen saturation, as well as the hematocrit, CRP, AST (aspartate aminotransferase) and ferritin [48]. Their nomogram showed an increased discriminatory power, with a C-index of 0.91 (95% CI, 0.85–0.97). Compared to these models, our prognostic nomogram was based on early laboratory parameters and incorporated two novel prognostic biomarkers: an angiogenetic growth factor, HB-EGF, and a representative cardio-microRNA, hsa-miR-195, which was previously reported as a risk prognostic factor for COVID-19 severity [16]. As shown in Figure 6, our decision curve had a wide threshold probability and a clinical net benefit higher than the two extreme cases of all-screening and no-screening strategies. Consequently, one can safely assume that the risk prediction model we have proposed is of clinical utility.

Nevertheless, our study has several limitations. Firstly, this study was conducted in a single hospital center, and our database did not include asymptomatic and mild COVID-19 patients; thus, we may have missed important data regarding the role of HB-EGF in this disease’s progression. Secondly, the number of patients included was limited due to the reduced compliance of COVID-19 patients; therefore, our results await external validation in larger cohorts. Thirdly, due to funding limitations, we could not perform an external validation of our results. Finally, our database did not provide a follow-up of the COVID-19 patients included in the study, and no inferences can be made regarding long COVID-19 development.

## 5. Conclusions

Altogether, our results defined previously ignored clinical correlations of HB-EGF plasma levels with COVID-19 symptomatology and risk factors, which might contribute to a better understanding of this disease’s clinical evolution. The risk prediction nomogram based on early biomarkers and the decision curve showed a good performance and might facilitate better-tailored patient management in clinical practice.

## Figures and Tables

**Table 1:** Demographics and baseline characteristics of the patients diagnosed with SARS-CoV-2 infection.

	COVID-19 Total (*n* = 75)	COVID-19 Moderate (*n* = 50)	COVID-19 Severe (*n* = 25)	*p*-Value Moderate vs. Severe
Age (Median, IQR)	60 (48.5–71)	53 (46.5–64)	71 (56–76)	0.0043 ^a^
Gender (Males/Females)	1.14	1.0	1.5	0.4681 ^b^
Hospitalization days (Median, IQR)	11 (8.5–15)	11 (8.25–14.75)	10 (9–15)	0.9978 ^a^
Associated pathologies *n* (%)				
Hypertension	36 (48.65)	20 (40.00)	16 (66.67)	0.0466 ^b^
Obesity	10 (13.51)	8 (16.33)	2 (8.00)	0.4788 ^b^
Diabetes	13 (17.11)	10 (19.61)	3 (12.00)	0.5265 ^b^
Cardiovascular pathology	30 (40.54)	14 (28.57)	16 (64.00)	0.0055 ^b^
Oncologic pathology	8 (10.81)	2 (4.08)	6 (24.00)	0.0155 ^b^
Chronic renal failure	6 (8.22)	2 (4.44)	4 (14.29)	0.1951 ^b^
Paraclinical Characteristics (Median, IQR)				
Leukocytes (×10^3^/µL)	6.23 (4.72–8.59)	6.04 (4.81–8.54)	6.96 (4.28–8.6)	0.8780 ^a^
Neutrophils (×10^3^/µL)	4.78 (2.65–7.05)	4.82 (2.66–6.44)	4.11 (2.76–7.45)	0.7400 ^a^
Lymphocytes (×10^3^/µL)	1.11 (0.69–1.57)	1.16 (0.84–1.53)	0.79 (0.52–1.69)	0.4356 ^a^
Platelets (×10^3^/µL)	230 (185.5–294)	226 (182.2–291.5)	235 (204–303)	0.6531 ^a^
Total Bilirubin (mg/dL)	0.42 (0.34–0.55)	0.44 (0.35–0.72)	0.42 (0.31–0.47)	0.2911 ^a^
Ferritin (µg/L)	527.4 (213.6–1210.5)	503 (161.1–1066.1)	581.5 (290.7–1473.2)	0.1805 ^a^
CRP (mg/L)	31.78 (6.37–75.88)	25.33 (5.07–72.27)	55.85 (15.42–109.06)	0.0827 ^a^
Fibrinogen (g/L)	5.16 (3.59–5.89)	4.71 (3.39–5.73)	5.47 (5.16–6.54)	0.0094 ^a^
PT (s)	11.6 (10.92–11.97)	11.4 (10.8–11.8)	11.8 (11.3–12.3)	0.0088 ^a^
Creatinine (mg/dL)	0.79 (0.64–0.92)	0.75 (0.63–0.91)	0.85 (0.79–1.03)	0.0269 ^a^
LDH (U/L)	249 (197–324.5)	249 (195.5–329.7)	246 (201–321)	0.9133 ^a^
D-Dimers (µg/mL)	0.45 (0.38–0.71)	0.45 (0.37–0.57)	0.50 (0.39–1.03)	0.0242 ^a^
Delta miR-195 (FC vs. control)	6.39 (5.34–7.69)	5.65 (4.95–6.47)	8.36 (7.73–9.24)	<0.0001 ^a^
HB-EGF (pg/mL)	358.3 (129.3–958.5)	185.6 (94.2–741.5)	483.5 (212.4–1220.5)	0.0371 ^a^
Therapy *n* (%)				
O_2_ supplementation	23 (30.67)	0 (0.00)	23 (92.00)	<0.0001 ^b^
Mechanical ventilation	10 (13.33)	0 (0.00)	10 (40.00)	<0.0001 ^b^

IQR: Interquartile range; M: Median; ^a^ Mann–U Whitney test; ^b^ Fisher’s exact test, FC (fold change expression).

**Table 2:** Symptomatology of COVID-19 patients stratified by severity.

Variables *n*, (%)	COVID-19 Total (*n*= 73)	COVID-19 Moderate (*n* = 45)	COVID-19 Severe (*n* = 28)	*p*-Value ^b^
Chills	47, (66.67)	30, (60.00)	17, (68.00)	0.6150
Running nose	11, (17.33)	7, (14.00)	6, (24.00)	0.3377
Sore throat	46, (61.33)	30, (60.00)	16, (64.00)	0.8051
Cough	42, (56.00)	28, (56.00)	14, (56.00)	>0.9999
Expectoration	20, (26.67)	12, (24.00)	8, (32.00)	0.5805
Dyspnea	49, (65.34)	26, (52.00)	23, (92.00)	0.0006
Thoracic pain	11, (14.67)	2, (4.00)	9, (36.00)	0.0005
Headache	30, (40.00)	22, (44.00)	8, (32.00)	0.4537
Myalgia	51, (68.00)	34, (68.00)	17, (68.00)	>0.9999
Vomiting	7, (9.33)	3, (6.00)	4, (16.00)	0.2127
Diarrhea	7, (9.33)	3, (6.00)	4, (16.00)	0.2127
Abdominal Pain	21, (28.00)	10, (20.00)	11, (44.00)	0.0542
Anosmia	9, (12.00)	9, (18.00)	0, (0.00)	0.025
Loss of taste	20, (26.66)	16, (32.00)	4, (16.00)	0.1734
Asthenia	36, (55.38)	26, (65.00)	10, (40.00)	0.0725 ^b^
Small patchy opacities	31, (41.33)	22, (44.00)	9, (36.00)	0.6210 ^b^
Large glass opacity	18, (23.00)	8, (16.00)	10, (40.00)	0.0422
Large consolidate opacity	16, (21.33)	6, (12.00)	10, (40.00)	0.0079
Diffuse infiltrate	6, (8.00)	1, (2.00)	5, (20.00)	0.0141

^b^ Fisher’s exact test.

**Table 3:** The multivariable binary logistic regression model for predicting COVID-19 severity.

Predictors	OR	OR 95% CI	|Z| Coefficient	*p*-Value
Delta miR-195	0.2164	0.07160–0.4352	3.482	0.0005
HB-EGF (pg/mL)	0.9994	0.9988–0.9999	2.082	0.0374
Age	1.032	0.9501–1.132	0.7311	0.4647
Fibrinogen (g/L)	0.5327	0.2151–1.082	1.593	0.1112
Prothrombin time PT (s)	0.3587	0.05469–1.242	1.313	0.1891
Creatinine (mg/dL)	0.4658	0.07241–5.133	0.8257	0.4090
D-Dimers (µg/mL)	0.7810	0.2619–1.084	0.7790	0.4360
Area Under Curve	0.9556		NPP (%)	89.47
95% CI	0.9063–1.000		PPP (%)	91.30
*p*-value	<0.0001		Tjur’s R squared	0.6756
Hosmer–Lemeshow test	statistic = 2.318			0.9697

OR—odds ratio, OR 95% CI—odds ratio’s 95% Confidence interval, |Z|—ratio of the estimated coefficient to its standard error, NPP—Negative Predictive Power, PPP—Positive Predictive Power.

## Data Availability

Data available in the Supplementary Materials.

## Supplementary Materials

The following supporting information can be downloaded at: https://www.mdpi.com/article/10.3390/biomedicines12020373/s1, File S1: Patients clinical data; File S2: Correlation analyses.
